# *IL28B* Genetic Variation Is Associated with Spontaneous Clearance of Hepatitis C Virus, Treatment Response, Serum IL-28B Levels in Chinese Population

**DOI:** 10.1371/journal.pone.0037054

**Published:** 2012-05-23

**Authors:** Xiaodong Shi, Yu Pan, Moli Wang, Dongsheng Wang, Wanyu Li, Tao Jiang, Peng Zhang, Xiumei Chi, Yanfang Jiang, Yanhang Gao, Jin Zhong, Bing Sun, Damo Xu, Jing Jiang, Junqi Niu

**Affiliations:** 1 Department of Hepatology, First Hospital of Jilin University, Changchun, China; 2 Department of Clinical Epidemiology, First Hospital of Jilin University, Changchun, China; 3 Institut Pasteur of Shanghai, Chinese Academy of Sciences, Shanghai, China; 4 Hospital of HepatologyBiliary of Jilin Province, Changchun, China; 5 Institute of Infection, Immunity and Inflammation, University of Glasgow, Glasgow, United Kingdom; 6 Fourth Hospital of Jilin University, Changchun, China; University of Tennessee Health Science Center, United States of America

## Abstract

**Background:**

The interleukin-28B gene (*IL28B*) locus has been associated with host resistance to hepatitis C virus (HCV) infection and response to PEG-IFN/RBV treatment in western populations. This study was to determine whether this gene variant is also associated with spontaneous clearance of HCV infection, treatment response and IL-28B protein production in Chinese patients.

**Methods:**

We genotyped *IL28B* genetic variations (rs12980275, rs8103142, rs8099917 and rs12979860) by pyrosequencing DNA samples from cohorts consisting of 529 subjects with persistent HCV infection, 196 subjects who cleared the infection, 171 healthy individuals and 235 chronic HCV patients underwent IFN/RBV treatment. The expression of IL-28B were measured by ELISA and RT-PCR.

**Results:**

We found that the four *IL28B* variants were in complete linkage disequilibrium (r2 = 0.97–0.98). The rs12979860 CC genotype was strongly associated with spontaneously HCV clearance and successful IFN/RBV treatment compared to the CT/TT. IL-28B levels in persistent HCV patients were significantly lower than subjects who spontaneously resolved HCV and healthy controls and were also associated with high levels of ALT (alanine aminotransferase) and AST (aspartate aminotransferase). IL-28B levels were also significantly lower in individuals carrying T alleles than CC homozygous.

**Conclusions:**

Thus, the rs12979860-CC variant upstream of *IL28B* gene is associated with spontaneous clearance of HCV, susceptible to IFN/RBV treatment and increased IL-28B levels in this Chinese population.

## Introduction

Hepatitis C virus (HCV) infection is a major health problem worldwide [1]. There are two distinct outcomes of an acute HCV infection; approximately 30% of HCV-infected individuals will spontaneously resolve HCV infection, developing robust protective immunity against reinfection. Among the remaining 70% patients, only half of them can be successfully treated with pegylated-interferon_α_ (pegIFN) plus ribavirin (RBV) [2]–[5] and the rest respond poorly to the treatment and will develop chronic infection which is the major cause of cirrhosis and hepatocellular carcinoma [6]–[8].

Thus, an understanding of the difference in host resistance to HCV infection and in response to treatment would be clinically important and may lead to novel therapeutic interventions.

The influence of viral genotype, load, sex and age on the natural course of HCV infection and outcomes have been evaluated with contradictory results [9]. Host innate and adaptive immunity also has a profound impact on HCV infection [10]. It has been suggested that chronic HCV infection may result from a impaired host immunity in particular cellular immune response against HCV [11]–[13]. It was recognized that genetic factor(s) may be responsible for the distinct consequences of HCV infection in different hosts [14], [15]. Interferons (IFNs) are the key cytokines produced by immune cells and play a pivotal role in natural host defense against HCV infection [16]. IFNα by far is the most effective means for clinical treatment of HCV infection. However, whether IFNs are the determining factor in the different outcomes of HCV infection is unknown until recently GWAS demonstrated that *IL28B* genetic variations are closely associated with host defence against HCV infection [17].

*IL28B* gene encodes cytokine IL-28B (IFNλ3) [18]. It belongs to the type III IFN family, also known as IFN-lamdas (IFNλs) which also includes IL-29 (IFNλ1) and IL-28A (IFN λ2) [19], [20]. The genes of IFNλs are clustered on human chromosome 19. IL-28B is the latest member of IFN family and plays a critical role in clearing viral infections [21], [22]. It is mainly produced by macrophages and dendritic cells (DCs) in response to the stimuli of viral proteins or toll-like receptor (TLR) agonists [23]. IL-28B plays an important role in antiviral responses including HCV infection [17]. IFNλs signal via common type III interferon receptors consisting of IL-10R2 and IL-28R subunit and trigger JAK-STAT (Janus kinase-signal transducers and activators of transcription) pathway to induce the expression of IFN-stimulate genes (ISGs) [16]. These ISG products can subsequently suppress a wide range of viral replications and protein synthesis including hepatitis B and C virus [24]. IFNλ receptors are expressed on a variety of cells, predominantly the liver and epithelial cells [19], [25], suggesting that they may have a profound impact on liver function.

Recently, several genome-wide association studies (GWAS) have independently demonstrated that *IL28B* variations, in particular rs12979860, are strongly associated with host natural defense against HCV infections in western populations [17]. They are also associated with HCV patients' response to pegylated-IFNα and RBV treatment in different populations [26], [27]. Thus, *IL28B* polymorphisms may represent determining factors in both host resistance/susceptibility to HCV infection and treatment, which may provide a genetic explanation for the different outcomes of HCV infection in distinct hosts. The gene frequency of C allele in rs12979860 is much higher in Chinese than Europe population (95.5% vs 68%) [17]. However, the influence and the cause-effect of the *IL28B* variant on HCV infection and IL-28B protein production in Chinese population is unknown.

The aim of this study was to explore the impact of the *IL28B* variations (rs12979860) on the clinical outcome of HCV infection, the treatment response and the mRNA and serum protein levels of IL-28B in a large Chinese Han population with HCV infection. A better understanding of the natural host immune defense against HCV will enhance our ability to treat HCV infection.

## Materials and Methods

### Patients

A total 896 Chinese Han individuals were enrolled in this study, all attended the “Epidemiological investigation of Hepatitis C virus infection in FuYu country of Jilin Province” in the First Hospital of Jilin University from 2009–2010. This study included 529 subjects with persistent HCV infection, 196 individuals who spontaneously cleared the virus, and 171 healthy controls. The persistent infection group included 340 male and 189 female HCV patients. All the subjects were human immune deficiency virus negative, anti-HCV positive, and HCV RNA positive in serum. The group showing spontaneous viral clearance comprised 101 males and 95 females who were anti-HCV positive and HCV-RNA negative. The healthy controls included 85 men and 86 women who had no medical history of any liver disease and were considered as representative of the normal frequencies of this study in the Chinese population.

Our study design is based on population-based sample. Healthy control population selection criteria: the same region, Han population, and no past liver disease history by routine physical examination, liver function, abdominal Ultrasound, no abnormal HBV and HCV tests. Equal number of men and women were in the healthy control group.

235 chronic HCV patients who underwent the IFN/RBV treatment regimen were also included in the study. These patients are receiving recombinant interferon-α2b (500,0000 IU, 3times/week, Bei Jing Kavin Technology Share-holding Co, Ltd. China) and oral ribavirin (<60 kg, 800 mg/day; 60–75 kg, 1000 mg/day; and >75 kg,1200 mg/day). Patients with decompensated liver disease, hepatoma, co-infection with hpatitis B virus or with human immunodeficiency virus, with apparent autoimmune hepatitis and alcoholic liver disease were excluded from this cohort. The definitions of response to treatment were undetectable serum HCV-RNA at 4 week after starting treatment as Rapid virologic response (RVR), at least two-log_10_ reduction of viral load at week 12 after starting treatment as early virologic response (EVR) and undetectable virus at week 12 after starting treatment as complete early virologic response (cEVR).

To participate in the study, written informed consent for all testing was obtained from all individuals, and the study protocol was approved by the hospital ethics committee of the First Hospital of Jilin University.

The participants completed questionnaires and underwent comprehensive medical examinations including liver ultrasound examination, anthropometric, blood pressure and blood tests. Blood samples were collected for biochemistry, liver function and serum lipids tests in the Clinical Laboratory of the First Hospital of Jilin University. The tests for biochemistry, liver function (ALT/AST) and serum lipids were performed using a Synchron LX®20 autoanalyser (Beckman Coulter, Brea, CA, USA). The levels of anti-HCV, HBsAg and human immune deficiency virus were measured using an Abbott ARCHITECT i2000SR and HCV-RNA levels were quantified using the Roche Amplicor HCV Monitor or Roche Taqman HCV test (Roche Diagnostics, Grenzach, Germany). HCV genotype was performed by multicolor fluorescence PCR with HCV RNA genotype kit (BioAssay Science & Technology Co. Ltd,China).

### IL28B genotyping

Human genomic DNA was extracted from peripheral blood cells by a genomic DNA purification kit (Promega Co., USA). Genotyping of the rs12979860, rs12980275, rs8103142 and rs8099917 was performed using a pyrosequencing method, according to the protocol of the manufacturer (PyroMark ID Pyrosequencing machine, QIAGEN). The primers for PCR were rs12979860: forward 5′-ATTCCTGGACGTGGATGGG TAC-3′, reverse 5′-biotin-AGCGCGGAGTGCAATTCA-3′; rs8099917: forward 5′-TTGTCACTGTTCCTCCTTTTGTTT-3′, reverse 5′-biotin-TGGGAGAATGCAAATGAGAGATA-3′; rs8103142: forward 5′- Biotin-AGAAGGTGAAGGGGCCACTA -3′, reverse 5′- CTGTGCCTTTGCTGTCTAGGA -3′; rs12980275: forward 5′-GCCAGTCTCAAAAGAACAAATGC-3′, reverse 5′-biotin-CTACCCCGGCAAATATTTAGACA-3′. Sequence primers for pyrosequencing analysis were rs12979860: 5′-AGCTCCCCGAAGGCG- 3′; rs8099917: 5′-TCCTTTCTGTGAGCAAT-3′; rs8103142: 5′-TGCTG A AGGACTGCA-3′; rs12980275: 5′-TGAGAGAAGTCAAATTCCT-3′.

### IL-28B ELISA

The serum IL-28B levels were determined with an ELISA Kit specific for IL-28B (Human λ3 IFN ELISA Kit, Catalogue number:I153-00, GBD Ltd. USA). The ELISA was carried out according to the manufacturer's instructions and the samples were measured in an ELISA-reader (Eppendorf® BioPhotometer, Germany) at 450 nm. The sensitivity of IL-28B ELISA was 1 pg/ml and has been validated in the range between 1–1000 pg/ml. The intraassay coefficient of variation was 3.7%, whereas that of the interassay variation was 5.2%.

### Quantitative real-time PCR

Peripheral blood mononuclear cells (PBMCs) were isolated from 74 subjects (48 persistent infection patients; 17 healthy individuals; 9 spontaneous clearance individuals) using Lymphocytes Separating Solution (DingGuo Biotechnology company, DH184-1). Total RNA extracted using trizol (Invitrogen) and cDNA was synthesized using random hexamers and SuperScript III Reverse Transcriptase (Invitrogen). Quantitative RT-PCR was performed using SYBR green (Realtime PCR MasterMix QPK-201, TOYOBO) on a 7300 Real-Time PCR System (Applied Biosystems, USA). The expression levels of IL28B mRNA were normalized to median expression of GAPDH. Primers used were: IL28A/B: forward 5′-CTG CCA CAT AGC CCA GTT CAA GT-3′, reverse 5′-ACT CTT CTA AGG CAT CTT TGG CCC-3′), GAPDH: forward 5′-GAA GGT GAA GGT CGG AGTC-3′, reverse 5′-GAA GAT GGT GAT GGG ATT TC-3′. The person performing gene expression analysis was blinded to the rs12979860 genotype.

### Statistics Analysis

Continuous variables were presented as mean (standard deviation) or median (interquartile), while categorical variables were expressed as frequencies (%). Differences between continuous variables were evaluated by means of the Student's t test or ANOVA. Differences between categorical variables were assessed using the Pearson Chi-square test. The chi-square G test “Goodness of Fit” was employed to verify whether the proportions of the polymorphism were distributed in the controls and HCV infection subjects in accordance with the Hardy-Weinberg equation. The existence of differences in allelic and genotypic frequencies between groups was assessed by Chi-square test. Stepwise logistic regression analysis with a forward approach was used to verify whether *IL28B* rs12979860 C/T polymorphism could be considered a predictor of the HCV spontaneous clearance. IL28B serum protein expression data was skewed distribution. Even after transforming the data was still not normal distribution so that we used non-parametric test methods for statistical analysis and the median and interquartile range for illustration. Prior to the analysis, the outlier (one) has been removed. The odds ratio (OR) with 95% confidence intervals (95%CI) were calculated using the same analysis. Pairwise linkage disequilibrium and r2 between SNPs were calculated by PLINK software. P-value <0.05 was considered a significant difference. Statistical analysis of data was performed using the SPSS software package 18.0 (SPSS Inc. USA).

## Results

896 Chinese subjects were involved in this study, including 529 subjects with persistent HCV infection, 196 subjects who cleared the infection and 171 healthy controls. The characteristics of the subjects and their demographic data, viral genotype, viral load and HBsAg status are summarized in Table 1.

**Table 1:** Characteristic of 896 study subjects.

Characteristic	Persistence	Clearance	Healthy control	*P*
	(n = 529)	(n = 196)	(n = 171)	
Mean age(years)(SD)	50.6(9.1)	51.7 (9.4)	39.6 (9.6)	<0.05
Male(%)	340 (64.3)	101(51.5) ▴	85(49.7) ?	<0.01
HBsAg status(% positive)	25(4.7)	23(11.7) ▴	none	<0.05
HCV load(×105 IU/ml)	11.2(0.8–31.2)	none	none	
HCV viral genotypes(%)^a^				
1b	163(57.6)	none	none	
2a	120(42.4)	none	none	
rs12979860				
Allele frequency(%)				
C	1008(95.3)#	383(97.7)	325(95.0)	<0.05
T	50(4.7)	9(2.3)	17(5.0)	
ALT(IU/L)	73.7(66.6)	35.0(30.5) ▴	24.0(23.9) #	<0.05
AST(IU/L)	57.2(45.3)	31.8(23.6) ▴	23.6(10.9) #	<0.05
GGT(IU/L)	87.8(66.9)	46.8(50.7) ▴	29.8(44.6) #	<0.05
TG(mmol/L)	1.4(0.9)	1.5(0.9)	1.5(0.8)	
TC(mmol/L)	4.5(1.0)	5.2(1.2)	4.9(1.0)	

a HCV viral genotypes was avaliable for 283 individuals in the persistent group.

▴ P<0.05 and

# P = 0.04 persistent vs clearence group;

# P<0.05, persistent vs healthy controls;

Quantitative variables are displayed as mean and SD; HCV load are displayed as median and quartile range. ALT, AST, GGT, TG, TC (alanine aminotransferase, aspartate aminotransferase, glutamyl transpeptidase, triglycerides and total cholesterol).

**Table 2:** Factors associated with virus spontaneous clearance by logistic regression analysis.

Factors	Comparison	Adjusted OR* (95% CI)	*P* value	Unadjusted OR§ (95% CI)	*P′* value
Sex	Male versus female	1.72 (1.23–2.41)	0.002	1.69 (1.21–2.36)	0.002
rs12979860 genotype	C/T+T/T versus C/C	2.13 (1.01–4.45)	0.04	2.07 (1.00–4.31)	0.05
HBsAg	Negative versus positive	2.85 (1.56–5.22)	0.001	2.68(1.48–4.84)	0.001

OR, odds ratio; CI, confidence interval.

* Included either rs12979860 genotype and sex, HBsAg with the following covariates: age, HCV load, viral genotype.

§ Included rs12979860 genotype and sex, HBsAg in the model without other covariates.

The frequencies of the key gene polymorphisms of *IL28B*; rs12979860, rs8099917, rs8103142 and rs12980275 were investigated in 303 subjects with persistent infection and 96 individuals who spontaneously cleared the virus (data not shown). The linkage disequilibrium (LD) and haplotype analysis with the four SNPs were performed. The result shows that these SNPs were in complete LD (D′ = 0.99–1; r^2^ = 0.97–0.98), therefore, the rs12979860 was selected as a tag SNP in the subsequent studies.

The distribution of the alleles of rs12979860 was in accordance with the Hardy-Weinberg equilibrium in this study (P = 0.51). We confirmed that the rs12979860 C allele frequency in this healthy Chinese population was 95%. The C allele was significantly lower in the persistent infection group (95.3%) than in the clearance group (97.7%) (Tab.1). However, the frequency of the T allele was greater among individuals of the persistent infection group than those of the clearance group (4.7% vs 2.3%; P = 0.04) (Tab. 1).

We further determined the association of the gene variants with spontaneous viral clearance by logistic regression (Tab. 2). Since there were fewer individuals with TT genotype of rs12979860 in this study we combined TT with TC genotype together for statistical analysis. As shown in Tab. 2, spontaneous HCV clearance was more common in subjects carrying the CC genotype than CT/TT genotype (P = 0.04, OR = 2.12, 95% CI = 1.01–4.42). The influence of sex and HBV co-infection was also determined by logistic regression (Tab. 2). Sex differences in the rate of spontaneous viral clearance were also observed; females appeared to be more effective in spontaneous viral clearance than males (P = 0.002). HBsAg status was also associated with spontaneous viral clearance (HBsAg negative vs positive: P = 0.001, OR = 2.85, 95% CI = 1.56–5.22 and male vs female: P = 0.002, OR = 1.72, 95% CI = 1.23–2.41). (Tab. 2).

The correlation between rs12979860 SNPs and virus load in chronic HCV subjects was further analysised. There was no association between IL-28B genotype and virus load in the whole patients (including genotype 1b or 2a), (data not shown). We did the test of interaction between viral genotype and *IL28B* genotype by Factorial design ANOVA, there is not statistical significance (P = 0.77).

**Figure 1:**
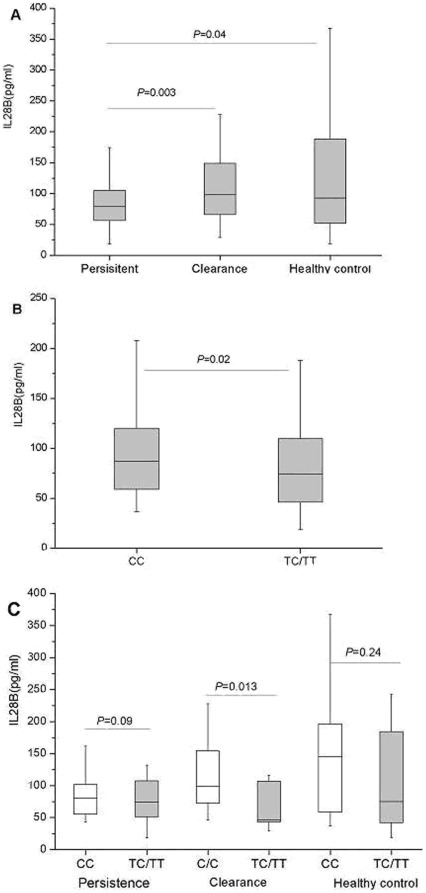
The association of serum levels of IL-28B with different outcomes of HCV infection and IL28B variants. IL-28B serum levels were determined by ELISA (A): compares the serum IL-28B levels among the persistent (n = 143), clearance (n = 56) and healthy control (n = 28) groups. (B): the association of IL-28B levels with rs12979860 alleles in all 227 subjects. (C): the IL28B serum levels in subjects carrying different alleles in different experimental groups. Data are median (quartile range) and are representative of two experiments.

The causative effect of rs12979860 variants on host resistance/susceptiblity to HCV infection is currently unknown. One possibility is that this gene variant upstream of the *IL28B* gene may affect IL-28B protein production. We then determined and compared the IL-28B levels in persistent, clearance and healthy control groups by ELISA. The IL-28B expression levels were comparable between the healthy control and clearance groups (Fig. 1A). However, the levels of IL-28B were markedly reduced in the persistent group compared to the spontaneous clearance and healthy control groups (Fig. 1A, persistent vs clearance, P = 0.003; persistent vs healthy control, P = 0.04). When stratified with respect to rs12979860 genotypes in overall subjects, IL-28B levels were significantly higher in CC homozygous than TT/TC home- or heterozygous (P = 0.02) (Fig. 1B). This was also the case in spontaneous clearance group (P = 0.013) but not in the healthy control or persistent groups (Fig. 1C). We also determined the mRNA levels of IL28A/B in PBMCs from different subjects by qRT-PCR. Our results showed that similar to the protein data in Fig. 1A and B, the levels of IL-28A/B mRNA were markedly reduced in the persistent group compared to the healthy control group (Fig. S2.A, persistent vs healthy control, P<0.001). When stratified with respect to rs12979860 genotypes, IL-28A/B levels were significantly higher in CC homozygous than TT/TC home- or heterozygous (P<0.001) (Fig. S2.B).

Transaminase levels indirectly reflect the liver function and host immune activation [28]. We next determined the levels of transaminase and their association with *IL28B* gene variants and the levels of serum IL-28B in chronic HCV subjects.

**Figure 2:**
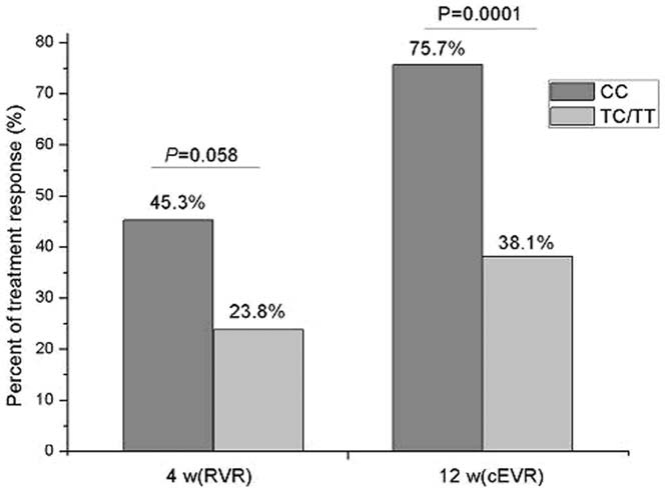
The association of rs12979860 with AST levels in chronic HCV patients. The AST levels were determined using a Synchron LX®20 autoanalyser. The AST levels were higher in patients carrying the T-allele. Data are mean±SD and representative of two experiments.

We assessed and compared the transaminase levels among the 896 subjects with persistent, cleared HCV infection or the healthy controls. We confirmed that the levels of ALT, AST and GGT were significantly higher in subjects with chronic HCV infection than those in individuals who cleared the infection and healthy controls (P<0.05) (Tab. 1). We further associated the levels of transaminase with *IL28B* variants in patients with persistent HCV infection (Fig. 2). The levels of AST were significantly higher in the patients carrying the rs12979860 T allele than the CC homozygous in the persistent group (P = 0.02, Fig. 2). However, no significant difference in their levels of ALT or GGT between CC and TC/TT carriers was found (data not shown).

The correlation between the levels of serum IL-28B and transaminase was also determined in 146 chronic HCV patients with high (ALT:>50 IU/ml; AST>40 IU/ml) or normal (ALT:≤50 IU/ml; AST:≤40 IU/ml) levels of transaminase. As shown in Fig. S1; there was a significant increase in IL-28B levels in chronic HCV patients with high levels of transaminase ALT and AST compared to those with normal levels (P = 0.001. Fig. S1A and B). In addition, there was no significant difference in IL-28B levels found between male and female subjects (P = 0.13) (data not shown). Further analysis revealed that there were also no significant differences in IL-28B levels between the HCV patients with high and low levels of HCV load, high and normal levels of total cholesterol (TC) or triglyceride (TG) or infected with 1b and 2a genotypes HCV (data not shown).

**Figure 3:**
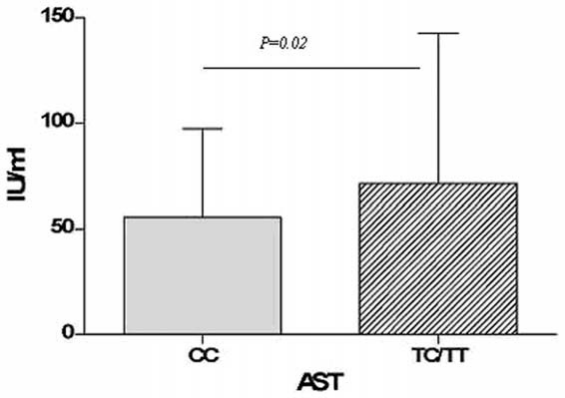
The association of IL28B genotype with response to IFN/RBV treatment. 235 chronic HCV patients underwent IFN/RBV treatment were involved in the study. the definition of RVR and cEVR was described in M&M. The IL28B CC genotype was significantly associated with RVR and cEVR.

Finally, we associated the *IL28B* polymorphisms with treatment response in 235 Chinese HCV subjects. The general information, basic demographic and virological features of the patients was displayed in Table 3. The RVR, EVR and cEVR rates to interferon-α2b and ribavirin treatment were 43.4%, 91.1% and 72.3%, respectively.the *IL28B* CC genotype increased the proportion of patients who attained cEVR, but not RVR (P>0.05), in Fig. 3, (cEVR = 75.7% for CC vs 38.1% for TC/TT, P = 0.0001). The HCV patients with *IL28B* CC genotype significantly increased cEVR, to a less extent, RVR to the treatment compared to the ones with TC/TT (Fig. 3). Furthermore, the multivariate logistic regression analysis further confirmed that the CC genotype was significantly associated with cEVR compared to TC/TT group (Table 4).

**Table 3:** Baseline characteristics of the patients treated with IFN/RBV.

Characteristic	Patients
	N = 235
Age(years), mean(SD)	49.9(7.4)
Gender, Male(%)	169(71.6)
BMI	23.8(2.6)
HCV genotype, n(%)*	
1b	127(54.0)
2a	85(36.0)
1b/2a	6(2.5)
HCV load (10^5^ IU/ml), median(range)	17(1.5–56)
HCV load>4×10^5^ IU/ml, n(%)	159(67.4)
rs12979860 CC/TC+TT, n(%)	214/21(91.1/8.9)
RVR, n(%)	102(43.4)
EVR, n(%)*	214(91.1)
cEVR, n(%)	170(72.3)

* Information of HCV genotype was unavailable for 17 patients and 4 patients who did not complete the treatment.

**Table 4:** Factors associated with RVR and cEVR by maltivariate Logistic regression.

Response	Factors	Comparison	OR (95% CI)	*P* value
RVR	Gender	female vs male	2.9(1.5–5.8)	0.004
	Baseline viral load(IU/ml)	<4×10^5^vs ≥4×10^5^	8.5(4.4–16.1)	<0.001
cEVR	rs12979860	CC vs TC/TT	6.1(2.2–17.2)	0.001
	HCV genotype	2a vs 1b	2.8(1.6–4.9)	<0.001
	Baseline viral load(IU/ml)	<4×10^5^vs ≥4×10^5^	5.1(2.1–12.1)	<0.001

OR (odds ratio); CI (confidence interval).

## Discussion

The data presented here demonstrates that the rs12979860 polymorphism of *IL28B* locus is closely associated with the outcomes of HCV infection in Chinese population. In our data, the normal frequency of rs12979860 T allele was 5% in healthy control of Chinese population. The frequency of T allele (rs12979860) was significantly higher among individuals with persistent HCV than those with cleared infection. Sex, ethnicity, and coinfections with hepatitis B virus affect spontaneous HCV clearance [29].

Even after adjustment for the gender and HBV infection in multivariate logistic regression, the CC and TC/TT of rs12979860 polymorphisms were still independently associated with spontaneous clearance and persistent HCV infection, respectively. The patients with TC and TT genotypes were two times less effective in their ability to clear HCV relative to patients with the CC genotypes (Tab. 2).

This is consistent with the recent GWAS, which shown that *IL28B* gene variants including rs8099917 are associated with the spontaneous HCV clearance in European and African American subjects [17], [26], [27], [30]–[32].

Intriguingly, the frequency of the protective rs12979860 CC-genotype is variable across the different ethnic groups; about 95% in Asia/Chinese population and approximately 55% and 51% in African and Europeans, respectively [17], [31]. Our result using a relative large cohort confirmed the result in Chinese population and suggested that *IL28B* polymorphism may represent a genetic advantage which confers anti-HCV immunity on the Asia/Chinese population. It may also provide a genetic explanation for the previous observations that while they seem to have similar HCV infection rate as other populations (about 3.2%), the Asian origin HCV patients appear to achieve better sustained virological response (SVR) rates when treated with PEG-IFN plus RBV compared to Caucasians [33]. It will be clinically important to reveal the detailed mechanism underlying the race influences on spontaneous virus clearance and antiviral treatment response.

We also demonstrated that the *IL28B* allele is associated with response to interferon and ribavirin treatment in Chinese patients with HCV infection. The patients carrying rs12979860 CC genotype are more likely to achieve cEVR which are the important indicator/predictors for the success of treatment in HCV patients [34]–[37].

The causative effect of rs12979860 polymorphisms on host protection against HCV infection is unknown. Since IL-28B possesses anti-viral activity and the SNPs were adjacent to the *IL28B* gene it has been suggested that the variants may associate with HCV infection by influencing IL-28B gene expression and protein production [38]. However, to date, little is known about the role of the gene variants in IL-28B production in HCV infection. Our results demonstrate that patients with chronic HCV infection had significantly lower mRNA and serum levels of IL-28B than subjects who spontaneously cleared the virus and the healthy controls. Individuals carrying protective rs12979860 CC genotype in general also tended to have higher IL-28B levels than those carrying the risk T-alleles in all subjects studied. Furthermore, the IL-28B levels were significantly higher in CC homozygous than the T homo- or heterozygous (P = 0.013) in the spontaneous clearance group. To support our finding, it has been reported that the rs8099917 T-protective allele was also associated with IL-28B mRNA expression in whole blood cells of healthy individuals [27]. These results suggest that *IL28B* variants can impact on IL-28B production which is correlated with host protection against HCV infection and that reduced IL-28B levels may be responsible for the chronicity of acute HCV infection.

The rs12979860 allele is 3 kb upstream from the *IL28B* locus which also contains several genes, including IL28A and IL-29 [19], [21]. It is likely that the SNP may also affect the function of other genes in the locus. Indeed, it has been reported that this variant is associated with increased serum IL-29 and IL-28A/B levels and the resolution of HCV infection [39], [40].

These results suggest that the variations upstream of *IL28B* gene may have an impact on the expression and production of all IFNλs, which may explain, at least in part, their profound association with the outcomes of HCV infection. Therefore, it is important to further elucidate the mechanism by which the gene variants regulate the expression of IFNλs in HCV infection.

Transaminases are the key clinical assessments for liver function and immune activation in HCV patients. We found the levels of transaminases (ALT, AST) were higher in patients with risk rs12979860-TC or TT than C/C genotype, especially AST. We also noted that the chronic HCV patients with high serum levels of ALT and AST aminotransferase also had relatively higher levels of serum IL-28B production (Fig. S1). The physiopathological significance of the observations and the relationship between IL-28B and aminotransferase levels in HCV infection is unknown. Since IL-28B is involved in anti-virus immunity we reason that higher aminotransferase and IL-28B levels may reflect the virus-host interaction and ongoing anti-HCV immune activation in the hosts and may also be an additional predicator for the outcomes of chronic HCV infection.

In conclusion, our results suggest that the SNP rs12979860 upstream of *IL28B* is associated with spontaneous clearance of HCV and treatment response in Chinese Han population. Furthermore, chronic HCV infection is associated with a reduced IL-28B production. Thus, IL-28B may represent a novel therapeutic agent against HCV and *IL28B* variations and IL-28B expression levels may help to predict the outcomes of HCV infection and treatment. Further research is needed to reveal the cause-effect of these polymorphisms on host protective immunity against HCV infection and the mechanism by which HCV downregulates IL-28B expression.

## Supporting Information

Figure S1 **The association of serum IL-28B levels with different levels of ALT/AST in chronic HCV patients.** Serum IL-28B levels were determined by ELISA and ALT/AST by a Synchron LX®20 autoanalyser. The IL28B levels were higher in patients with high ALT (>50 IU/ml) (A) or AST (>40 IU/ml) (B) than those with normal ALT (≤50 IU/ml) or AST (≤40 IU/ml). Data are median (quartile range) (n = 147) and representative of 3 experiments.
(TIF)

Figure S2 **The relative expression levels of IL28 with different outcomes of HCV infection and IL28B variants by qRT-PCR.** (A): compares the IL-28A/B expression among the persistence (n = 48), clearance (n = 9) and healthy control (n = 17) groups. (B): the association of IL-28A/B expression with rs12979860 alleles in all 74 subjects. Data are median (quartile range) and are representative of two experiments.
(TIF)
