# Supplementary figures and images for: IL28B Genetic Variation Is Associated with Spontaneous Clearance of Hepatitis C Virus, Treatment Response, Serum IL-28B Levels in Chinese Population
Source: PLoS One. 2012 May 23;7(5):e37054. doi: 10.1371/journal.pone.0037054 (PMC3359351; doi:10.1371/journal.pone.0037054)

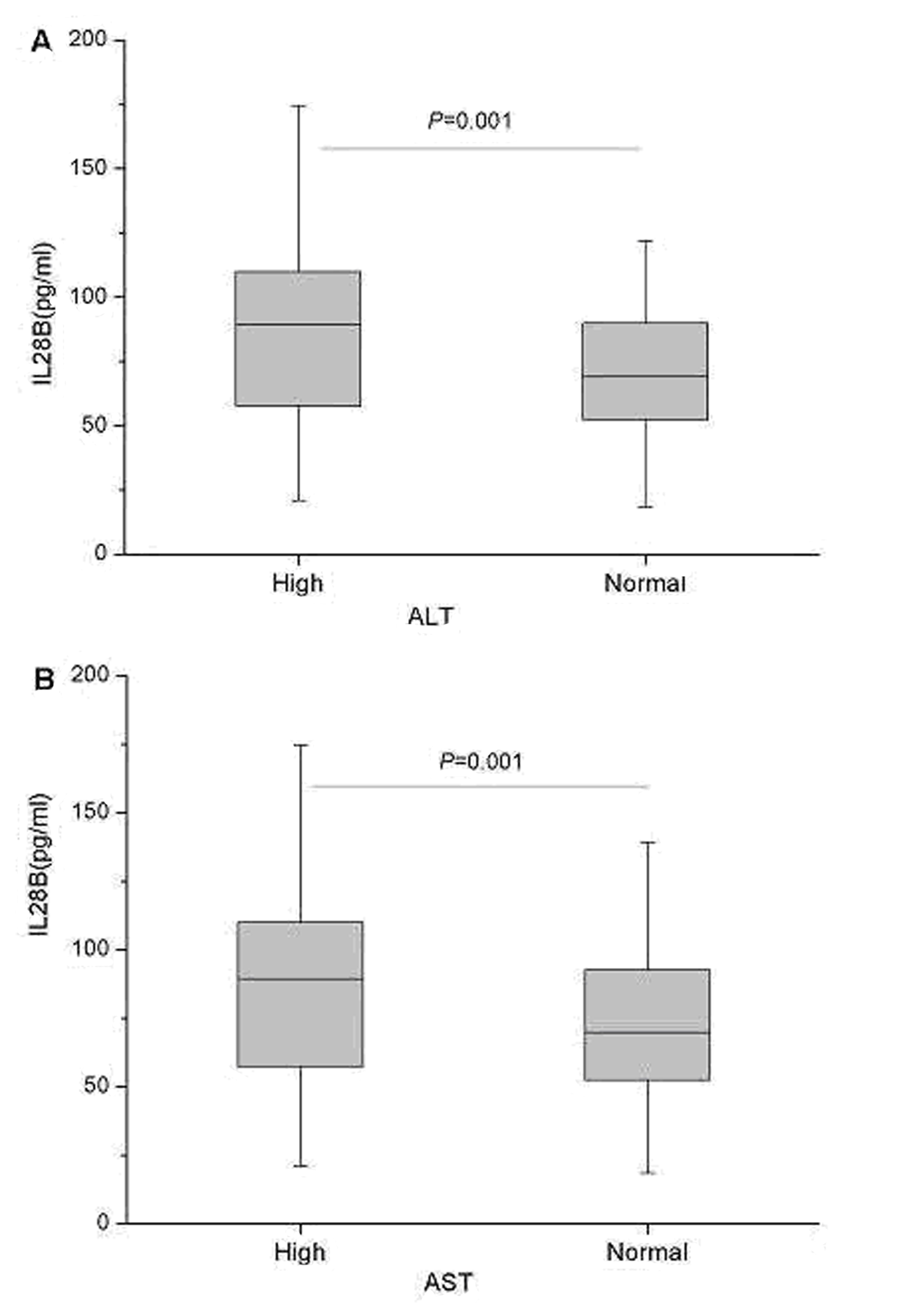

Supplement: Figure S1 — The association of serum IL-28B levels with different levels of ALT/AST in chronic HCV patients. Serum IL-28B levels were determined by ELISA and ALT/AST by a Synchron LX®20 autoanalyser. The IL28B levels were higher in patients with high ALT (>50 IU/ml) (A) or AST (>40 IU/ml) (B) than those with normal ALT (≤50 IU/ml) or AST (≤40 IU/ml). Data are median (quartile range) (n = 147) and representative of 3 experiments. (TIF) [file pone.0037054.s001.tif]

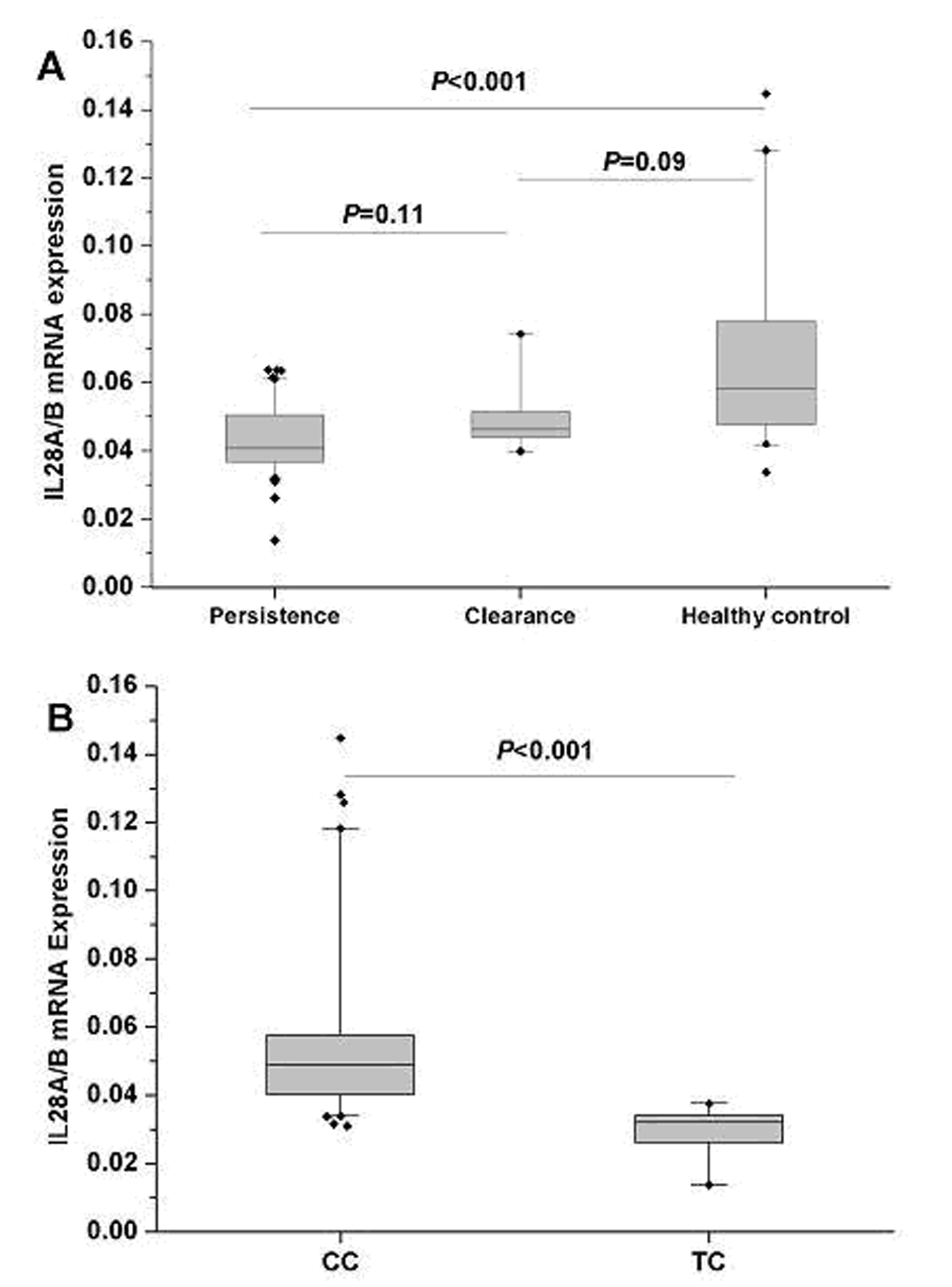

Supplement: Figure S2 — The relative expression levels of IL28 with different outcomes of HCV infection and IL28B variants by qRT-PCR. (A): compares the IL-28A/B expression among the persistence (n = 48), clearance (n = 9) and healthy control (n = 17) groups. (B): the association of IL-28A/B expression with rs12979860 alleles in all 74 subjects. Data are median (quartile range) and are representative of two experiments. (TIF) [file pone.0037054.s002.tif]
